# Utility of quantitative whole-body autoradiography (QWBA) and oxidative combustion (OC) analysis in the assessment of tissue distribution of [^14^C]Mefuparib (CVL218) in SD and LE rats

**DOI:** 10.1371/journal.pone.0315223

**Published:** 2024-12-05

**Authors:** Xinmei Li, Feiyu Wang, Xinyue Zhang, Xiaokun Shen, Chunhao Yang, Yuandong Zheng, Xingxing Diao

**Affiliations:** 1 Department of Pharmacy, Fuwai Yunnan Hospital, Chinese Academy of Medical Sciences/Affiliated Cardiovascular Hospital of Kunming Medical University, Kunming, China; 2 School of Chinese Materia Medica, Nanjing University of Chinese Medicine, Nanjing, China; 3 Chinese Academy of Sciences, Shanghai Institute of Materia Medica, Shanghai, China; 4 Convalife (Shanghai) Co., Ltd., Shanghai, China; Ural Federal University named after the first President of Russia B N Yeltsin Institute of Physics and Technology: Ural’skij federal’nyj universitet imeni pervogo Prezidenta Rossii B N El’cina Fiziko-tehnologiceskij institut, RUSSIAN FEDERATION

## Abstract

**Background:**

In tissue distribution studies of radiopharmaceuticals, quantitative whole-body autoradiography (QWBA) and oxidative combustion (OC) analysis are the two important methods that have not been compared using the same drug. Sprague-Dawley (SD) and Long-Evans (LE) rats, both of which are commonly used rodents in tissue distribution studies, have also not been compared using the same drug. Comparative studies are important for aiding the selection of appropriate experimental methods and animals.

**Methods:**

To evaluate the tissue distribution of [^14^C]Mefuparib (CVL218) in rats and assess its clinical safety, QWBA and OC analysis were used. The differences between the two methods were noted. Comparisons between the tissue distribution results of LE and SD rats were also done.

**Results:**

The QWBA and OC distribution analysis showed that [^14^C]CVL218-related radioactivity could be distributed in 19 tissues. For 89.47% of the tissues, no significant differences were noted between the two methods. There were also no differences in the pharmacokinetics data for plasma and brain homogenates between LE and SD rats. However, the pharmacokinetics data for liver and kidney homogenates were seven-fold higher in LE rats than in SD ones.

**Conclusions:**

Both the OC and QWBA methods revealed that [^14^C]CVL218 could be widely distributed in the tissues of rats. The OC had a lower limit of quantification while QWBA provided a more comprehensive analysis of [^14^C]CVL218 distribution. More safety was associated with using LE rat data to estimate the dosimetry of [^14^C]CVL218 for the whole-body, for human radiolabeled mass balance studies.

## 1. Introduction

Tissue distribution studies are essential for the human dosimetry assessment of radiopharmaceuticals in human radiolabeled mass balance studies [1–4]. Quantitative whole-body autoradiography (QWBA) and oxidative combustion (OC) analysis are the two methods that are commonly used in these studies. The QWBA method involves analyzing whole body sections of animals after drug administration using the phosphor imaging technology [5–7]. The concentrations of drug-related substances (both parent drug and metabolites) in the tissue regions are then analyzed against a standard calibration curve in the phosphor images. The OC method involves evaluating the concentrations of drug-related substances in animal tissues using oxidation combustion technology and liquid scintillation technology [8–10]. Ideally, selecting only one of the QWBA and OC methods for tissue distribution studies is preferred as it is cost-effective. However, there haven’t been studies that reported the similarities and differences between the two methods. This study involved tissue distribution studies of the two methods in Long-Evans (LE) male rats and compared the two methods. The results would provide researchers with a basis for selecting the appropriate method of their scientific investigations.

Sprague-Dawley (SD) rats and LE rats are two commonly used animal strains in tissue distribution studies. Some studies suggested that the difference in tissue distribution between SD and LE rats mainly lies in the distribution of melanin in LE rats, as evidenced by the eyes and pigmented skin [11]. Plasma pharmacokinetics and central nervous system tissue distribution studies suggested that there may be no statistical difference between the LE and SD rats [12–14].

Mefuparib (CVL218) is a second-generation PARP inhibitor. Previous studies revealed that [^14^C]Mefuparib-related radioactivity could be widely distributed in various tissues. No significant differences were found in the concentrations in the plasma and brain tissue homogenates of LE and SD rats at the same time points. There are no available reports on differences in the distribution of non-pigment organs between SD and LE rats. This study focused on the differences in the distribution of major metabolic organs between SD rats and LE rats, with more focus on the liver and kidney. The purpose was to provide guidance that investigators could use for selecting animal strains for tissue distribution studies. This was done to provide more reliable assessment data and reduce the risk of Phase I clinical studies.

## 2. Materials and methods

### 2.1. Chemicals and reagents

The sources of [^14^C]CVL218 (99.65%, 56.43 mCi/mmol), CVL218 (99.60%), the liquid scintillation cocktail, and alkaline liquid scintillation cocktail were the same as those in the literature [8].

### 2.2. Animals

The approval, sources, welfare, and feeding conditions of male SD and LE rats were the same as those reported in the literature [8–10]. This study protocol was approved by the Animal Ethics Committee of the Shanghai Institute (IACUC NO.2022-07-DXX-06) of Materia Medica, Chinese Academy of Science (Shanghai, China).

### 2.3. Dose preparation and dosing

The intragastric administration (i.g.) solution consisted of [^14^C]CVL218 and CVL218. The concentration of the solution was 2 mg/mL (10 μCi/mL). The dosage of a single i.g. administration was 20 mg/kg (100 μCi/kg, 10 mL/kg). The radioactivity of the dosing formulation was the same as that reported in the literature [8].

### 2.4. Quantitative whole-body autoradiography (QWBA) analysis

At 0.5, 2, 8, and 48 h post dose, four male LE rats were collected and their whole blood was centrifuged to obtain the plasma. The plasma was assayed using the liquid scintillation technique. After collecting the blood from LE rats, the animals were euthanized using a CO_2_ overdose before they were frozen. Whole animals were embedded into blocks of 2% aqueous carboxy methyl cellulose. Whole-body sagittal plane sections were obtained using a microtome (Leica CM3600 Cryomacrocut, Nussloch, Germany). Appropriate sections were selected at various levels of interest and the tissue samples were collected for OC analysis. The 35 μm sections were collected on adhesive tape. The sections were cut and freeze-dried at -20°C for at least 72 h, before they were exposed alongside calibration standards of ^14^C-blood (0.001, 0.003, 0.005, 0.010, 0.030, 0.053, 0.101, 0.298, 0.507, 0.983, 2.911, and 5.291 μCi/g). The ^14^C-sensitive phosphor imaging plates (Cytiva, Uppsala, Sweden) and thin plastic wrap covered sections were placed in light-tight exposure cassettes (Cytiva, Uppsala, Sweden) at room temperature, for three days. The tissue distribution of radioactivity ([^14^C]CVL218) was determined using the Amershan Typhoon image acquisition system (Cytiva, Uppsala, Sweden) along with the Amershan Typhoon Scanner (Cytiva, Uppsala, Sweden) and AIDA image analyzer v.5.1 (Elysia S.A., Angleur, Belgium) software.

### 2.5. Oxidative combustion (OC) analysis in LE and SD rats

Tissue samples, including those from the plasma, brain, liver, and kidney were collected from 12 male LE rats (0.5, 2, 8, 48 h) and the same number of male SD rats (0.5, 2, 8, 24 h) for oxidative combustion (OC) analysis [8]. The collection method and storage conditions were the same as those reported in literature [8]. The total radioactivity was also consistent with what is reported in literature [8–10, 15, 16].

### 2.6. Statistical analysis

Plasma radiopharmaceutical parameters were calculated using the WinNonlin 7.0 Software (Pharsight Corporation, Mountain View, CA, USA) via noncompartmental modeling. The experimental data were expressed as mean ± standard deviation. Statistical analyses between the two groups were done with a t-test, using the GraphPad Prism 6 software (San Diego, CA, USA). Significant differences were considered when *P* < 0.05.

## 3. Results

### 3.1. Comparisons between QWBA and OC analyses

Based on the results from quantitative whole-body autoradiography (QWBA) method, radioactive-related substances were detected in all the 19 tissues that were observed at 0.5, 2, 8, and 48 hours after drug administration, with very high concentrations in the small intestine, stomach, large intestine, prostate, and bladder (Table 1). At 48 h post-dose, the radioactivity in most tissues had dropped to BLQ (198 ng eq./g). However, trace radioactivity concentrations were recorded for the eye (7529 ng eq./g), skin (1585 ng eq./g), liver (991 ng eq./g), adrenal glands (793 ng eq./g), testis (594 ng eq./g), kidney (396 ng eq./g), lung (198 ng eq./g), heart (198 ng eq./g) and small intestine (198 ng eq./g). In general, high concentrations of radioactivity were found mainly in tissues involved in the distribution and excretion of drug-related substances.

**Table 1:** Concentration of [^14^C]CVL218 in tissue of interest from male LE rats after a single 20 mg/kg (100 μCi/kg) intragastric administration by quantitative whole-body autoradiography (QWBA) and oxidative combustion (OC) analysis.

Mmethod tissue	Quantitative whole-body autoradiography (QWBA)					Oxidative combustion (OC)					OC:QWBA AUC ratio
	Concentration (ng eq./g)				AUC_0-48h_	Concentration (ng eq./g)				AUC_0-48h_	
	0.5 h	2 h	8 h	48 h	(ng eq./g h)	0.5 h	2 h	8 h	48 h	(ng eq./g h)	
eye	2180	19418	36458	7529	1064131	4391	24065	52239	38940	2074921	0.51
brain	793	2576	594	BLQ	24124	2438	3499	1538	52	51984	0.46
skin	1783	7133	991	1585	83022	2053	7992	4666	5706	253468	0.33
fat	198	594	198	BLQ	6985	1481	3553	989	131	40165	0.17
muscle	2378	3765	594	BLQ	30167	1757	4430	1418	40	51776	0.58
lung	11096	21598	5746	198	228212	32288	41960	13752	194	509803	0.45
heart	4755	2378	1585	198	54093	5822	8012	2043	65	84169	0.64
liver	39034	25362	8520	991	349922	36965	39011	13700	1988	538114	0.65
spleen	8520	18626	6142	BLQ	219642	10689	19873	6391	117	234547	0.94
pancreas	396	991	396	BLQ	13226	9009	13105	3094	107	131464	0.10
kidney	21201	23183	14861	396	457861	33679	33041	12017	849	450962	1.02
adrenal gland	19220	19220	9115	793	316782	22499	16211	5212	408	211335	1.50
bladder	44186	69350	1585	BLQ	340708	2542	8552	4506	89	140038	2.43
prostate	24966	7926	114527	BLQ	2688811	2423	9624	4234	65	137196	19.60
thymus	2378	9115	4755	BLQ	145933	3524	9441	4429	42	141633	1.03
testis	793	3170	4359	594	124831	896	3638	4209	58	112501	1.11
stomach	2774	106403	1387	BLQ	433687	50631	19735	5027	317	246599	1.76
small intestine	177933	54291	2774	198	449291	70882	27177	10967	222	429469	1.05
large intestine	3368	9709	145239	BLQ	3380284	5516	7172	6519	541	193155	17.50
plasma	5484	4986	1535	111	61687	3208	7224	2975	113	100970	0.61
blood	3567	3963	1189	BLQ	45771	3953	5270	2035	95	72403	0.63

BLQ, below limit of accurate quantification (<198 ng eq./g)

Based on the results from oxidative combustion (OC) method, radioactive-related substances were detected in all the 19 tissues that were observed. This indicated that [^14^C]CVL218 was widely distributed in the tissues of LE rats (Table 1). At 48h post-dose, the radioactivity concentration was mainly detected in eye (38940 ng eq./g), skin (5706 ng eq./g), liver (1988 ng eq./g), kidneys (849 ng eq./g), large intestine (541 ng eq./g), adrenal glands (408 ng eq./g), stomach contents (317 ng eq./g), small intestine (222 ng eq./g) and lungs (194 ng eq./g). The OC/QWBA ratio of 89.47% of the tissues (except for prostate and large intestine) was less than 3.0. This suggested that the results of radioactive-related substances were not significantly different between the two methods.

**Fig 1:**
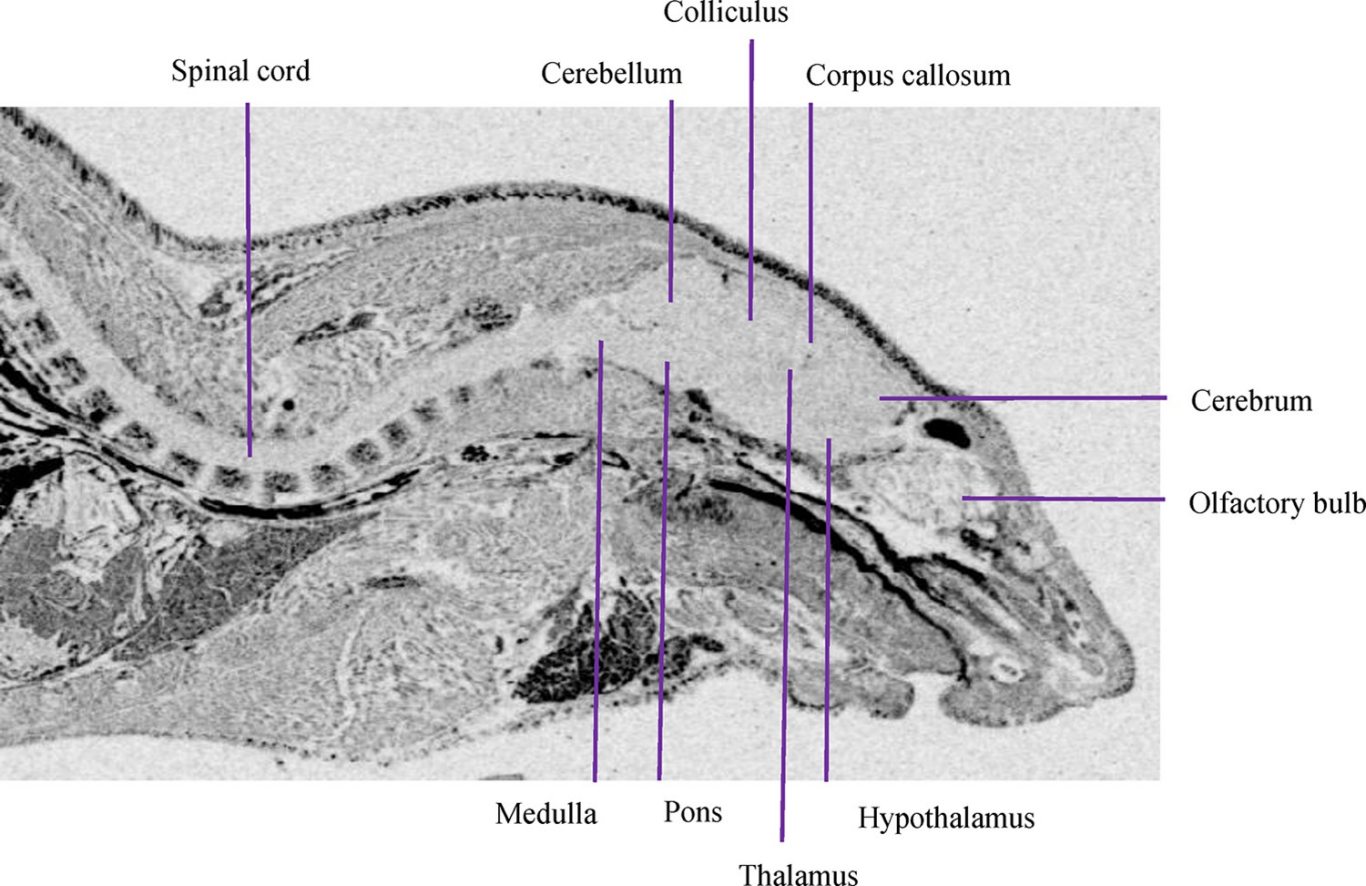
Central nervous system autoradiogram of the radioactivity distribution in a male LE rat at 2 h post-dose following a single i.g. administration of [^14^C]CVL218 at a target dose of 20 mg/kg (100 μCi/kg).

When the QWBA method was used, the results showed that [^14^C]CVL218 was uniformly distributed in 10 substructures of the central nervous system (CNS) (Table 2). When the OC method was used, however, we were unable to differentiate between substructures of the CNS and were only able to detect the concentration of drug-related substances in brain homogenates (Table 3). The corpus callosum had the highest distribution concentration 2 h after administration (Fig 1). The concentration of all substructures was below the limit of accurate quantification (<198 ng eq./g) 48 h post-dose. This showed that the drug was eliminated from the substructures of the CNS.

**Table 2:** Concentration of [^14^C]CVL218 in brain tissue of interest from male LE rats after a single 20 mg/kg (100 μCi/kg) intragastric administration by quantitative whole-body autoradiography (QWBA).

Male brain tissue	Concentration (ng eq./g)				AUC_0-48h_
	0.5 h	2 h	8 h	48 h	(ng eq./g h)
Olfactory bulb	198.14	2179.58	396.29	BLQ	17486.19
Cerebrum	198.14	2179.58	594.43	BLQ	22043.50
Corpus callosum	198.14	2774.01	396.29	BLQ	19715.31
Colliculus	594.43	2179.58	198.14	BLQ	13325.17
Cerebellum	396.29	2179.58	396.29	BLQ	17684.33
Thalamus	396.29	2377.73	594.43	BLQ	22984.68
Hypothalamus	198.14	2179.58	594.43	BLQ	22043.50
Pons	594.43	2179.58	594.43	BLQ	22439.79
Medulla	396.29	2377.73	396.29	BLQ	18427.37
Spinal cord	BLQ	2179.58	792.58	BLQ	26402.66

BLQ, below limit of accurate quantification (<198 ng eq./g)

**Table 3:** Pharmacokinetics parameters of the total drug-related substances in the plasma, brain homogenate, liver homogenate, and kidney homogenate of LE rats and SD rats after a single intragastric administration of [^14^C]CVL218.

Matrix	LE rats								SD rats								AUC_0-∞_ ratio (LE/SD)
	λz	*t*_1/2_	*T*_max_	*C*_max_	AUC_0-48h_	AUC_0-∞_	AUC__%Extrap_	MRT_0-∞_	λz	*t*_1/2_	*T*_max_	*C*_max_	AUC_0-24h_	AUC_0-∞_	AUC__%Extrap_	MRT_0-∞_	
	(1/h)	(h)	(h)	(ng eq.·mL^-1^)	(ng eq.·h·mL^-1^)	(ng eq.·h·mL^-1^)	(%)	(h)	(1/h)	(h)	(h)	(ng eq.·mL^-1^)	(ng eq.·h·mL^-1^)	(ng eq.·h·mL^-1^)	(%)	(h)	(%)
Plasma	0.09	7.89	2.00	7223.67	100967.75	102292.34	1.26	7.70	0.19	3.72	1.50	5968.89	53735.39	54278.70	1.05	5.44	1.88
Brain	0.09	7.89	1.50	3759.33	51980.83	52598.99	1.26	7.56	0.20	3.52	1.50	2629.56	17546.55	17689.97	0.84	4.63	2.97
Liver	0.06	11.97	1.50	51007.67	538120.67	572813.06	6.00	12.18	0.15	4.79	1.50	11725.50	77155.62	79598.12	3.22	5.62	7.20
Kidney	0.07	9.46	1.00	39665.67	450975.42	462561.87	2.72	8.77	0.16	4.39	1.50	8037.40	60827.59	62144.08	2.23	5.60	7.44

### 3.2. Oxidative combustion (OC) analysis in LE and SD rats

**Fig 2:**
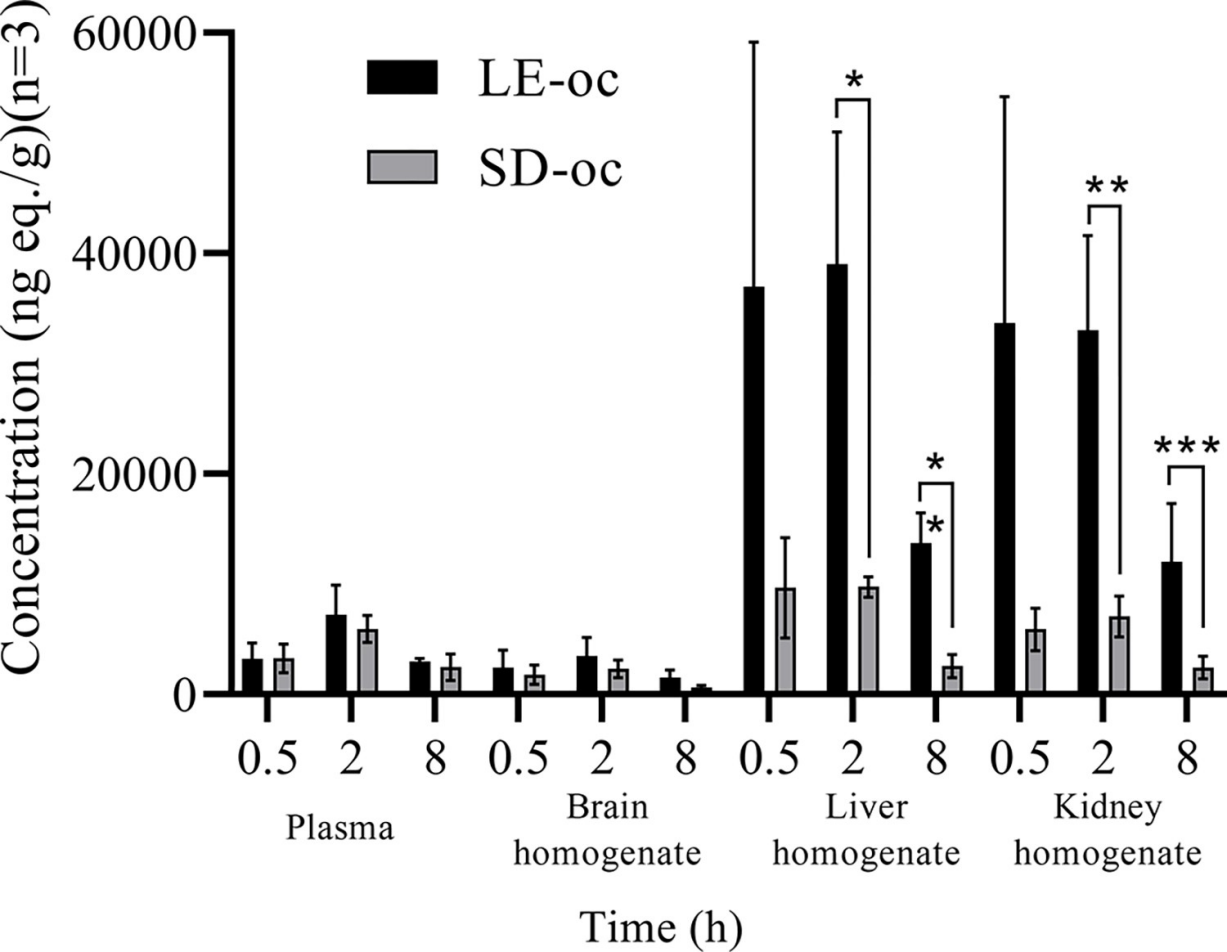
Concentration of [^14^C]CVL218 in the plasma, brain homogenate, liver homogenate, and kidney homogenate of LE rats and SD rats using oxidative combustion (OC) analysis at 0.5, 2, and 8 hours post-dose following a single i.g. administration. *: There was a significant difference (P<0.05).

There was no significant difference (P>0.05) in the concentrations of drug-related substances in the plasma and brain homogenates of SD and LE rats at 0.5, 2, and 8 hours post-dose (Fig 2). The AUC_0-48h_ results that were calculated using WinNonlin showed that the pharmacokinetic outcomes in the plasma and brain homogenates were less than 3.0. This indicated that there was no significant difference between SD and LE rats, based on these homogenates (Table 2).

The concentrations of drug-related substances in the liver and kidney homogenates of SD and LE rats at 0.5 h post-dose were not statistically significantly different (P>0.05)(Fig 1). At the 2- and 8-hour points after drug administration, there was a significant difference (P<0.05) in the concentrations of drug-related substances in the liver and kidney homogenates of SD and LE rats. The PK parameters calculated using WinNonlin showed that the total drug-related substances in the liver homogenate were 7.20 higher in LE than SD rats. Moreover, the AUC_0-∞_ in the kidney homogenate was 7.44 higher in LE than SD rats (Table 2).

## 4. Discussion

### 4.1. Comparison of QWBA and OC analysis

Since the introduction of phosphor imaging plates and their scanners, quantitative whole-body autoradiography (QWBA) has been considered a possible alternative to the oxidative combustion (OC) method. Recent research shows that the accuracy of the QWBA method is comparable to that of the OC method. In this study, both methods (QWBA and OC) were used to investigate different concentrations of [^14^C]CVL218 in rat tissues. There was no significant difference in the data analyzed by the OC and QWBA methods after [^14^C]CVL218 had been administered to most tissues. This is because the OC:QWBA values of the majority of tissues and organs were less than three. The largest differences between the two methods were in radioactivity concentrations in the prostate and large intestine, with OC/QWBA ratios of 19.60 and 17.50, respectively. This difference may be due to signal enhancement caused by excreta, which accumulates differently in the large intestine and prostate. Additionally, continuous reabsorption and enterohepatic circulation may result in lower signals in the large intestine. It is important to note that these are objective observations and not subjective evaluations. The OC/QWBA ratios of the prostate and colon may differ. Moreover, the QWBA method collects data from only one rat per time point, and this may contribute to the differences in OC/QWBA ratios for the prostate and large intestine between individual animals.

OC analysis after homogenization mainly reflects the overall distribution of drug-related substances in the organ. The OC method captures the ^14^CO_2_ produced by combustion using the alkaline scintillation solution on homogenated tissues. The lower limit of quantification (1.0 ng eq./g) of data obtained using the OC method is generally lower than that of the QWBA method (198 ng eq./g). This is attributed to the high sensitivity of the liquid scintillation technique. At 48 hours, the OC method measured the lowest tissue radioactivity at 40 ng eq/g. The OC method is more effective than the QWBA method in observing detailed information for low tissue concentrations. In addition to metabolic information, the OC method provided unique pharmacokinetic parameters for radiolabeled CVL218 in the plasma, brain homogenate, kidney homogenate, and liver homogenate. In addition, the homogenized samples can be used to analyze the tissue metabolite composition when the OC method is used. However, the QWBA method does not permit further metabolite analysis and identification as it is based on rat sectioning. However, the QWBA method does not permit further metabolite analysis and identification as it is based on rat sectioning. However, the disadvantage associated with the homogenate method is the large number of animals required when sampling time is crowded. This labor-intensive traditional method is limited by the complex structure of the tissue substructures such as the CNS, making it difficult to separate the individual fine subdivisions during the actual procedure [17].

**Fig 3:**
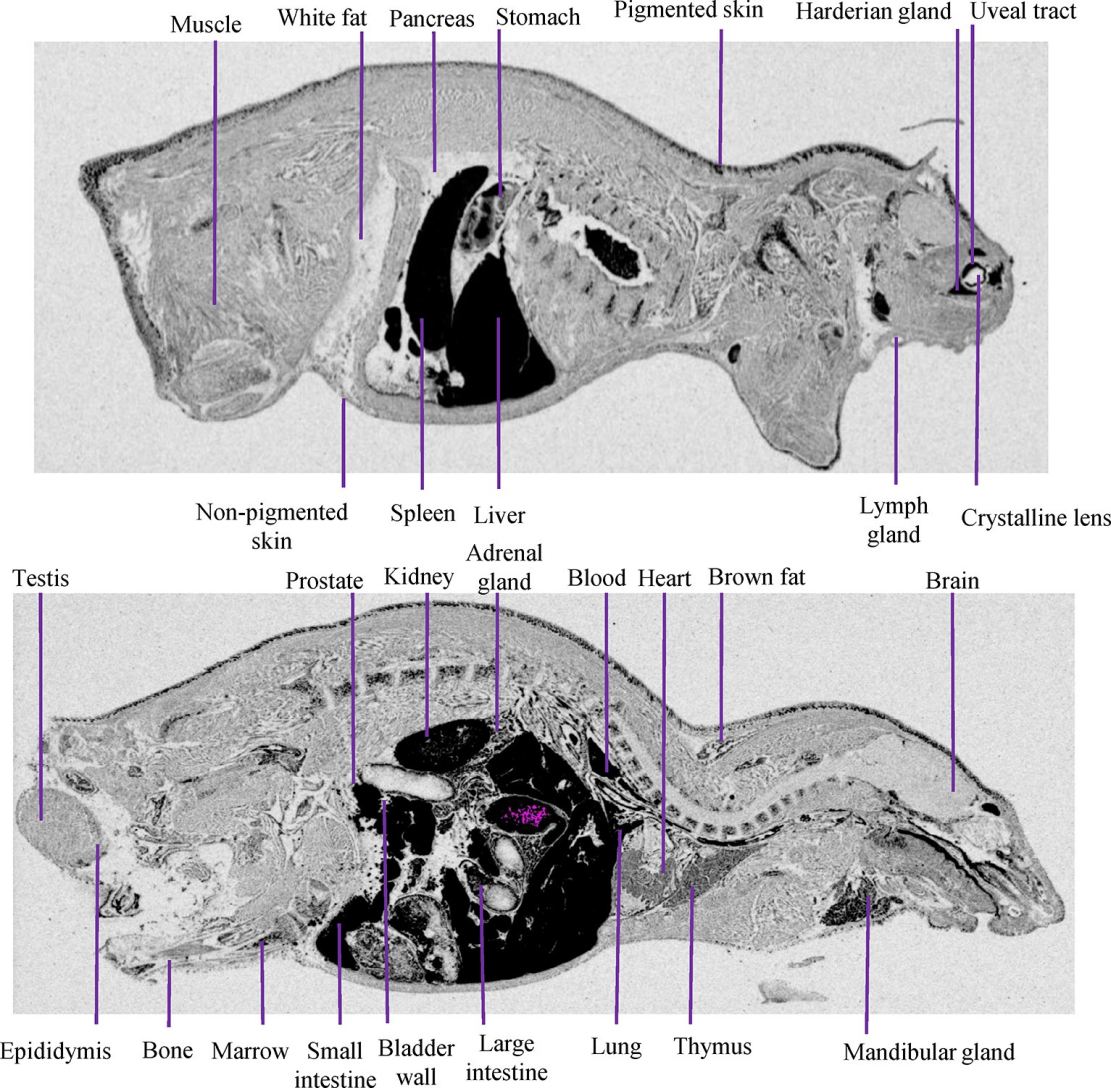
Whole-body autoradiogram of the radioactivity distribution in a male LE rat at 2 h post-dose following a single i.g. administration of [^14^C]CVL218 at a target dose of 20 mg/kg (100 μCi/kg).

The QWBA method solves this problem by allowing a visual comparison of the differences in the drug distribution of radioactive substances in different tissues and organs simultaneously, while using fewer animals. With this method, the differences in drug concentration distribution across various tissues and organs can be visualized while preventing contamination of organs by body fluids, which is a common issue during tissue dissection. The benefits of using the QWBA method are clear. Table 1 presents the drug concentrations obtained through the QWBA and OC methods at different time points in the tissues of LE rats. Fig 3 provides insight into CVL218 distribution in the glands. However, the Harderian and Mandibular glands were not captured in the OC analysis. QWBA found drug distribution in these two tissues. The disadvantage of this method is that it has relatively higher requirements with regard to the experimental site, selection of tissue sections, and the exposure environment for the sections. Additionally, the current method has an exposure time of three days, showing that it’s time-consuming.

We suggest the development of new imaging agents to save time, while also improving sensitivity, and specificity. QWBA is currently the most commonly used method for analyzing healthy rodents. There is even more potential for its application in studies on oncology and metabolic disorders. Combining the QWBA technique with other imaging techniques such as PET/CT, ultrasound, and magnetic resonance imaging could improve the accuracy of drug-targeted therapies. Moreover, its potential application in clinical settings could be further explored.

### 4.2. Advantages of LE rats: Pigment

Compared with the SD rat with red eyes and white skin, the LE rat has black eyes and skin on its head and back. This LE rat strain attained attention as a mature rodent model for research on tissue distribution. It has been recognized by the Center for Drug Evaluation of the National Medical Products Administration (CDE, China) and the Food and Drug Administration (FDA, USA). There are two advantages of this strain. First, the retinal pigment epithelium in LE rats is similar to that of the human retina, making it an ideal animal model for studying drug accumulation in the eyes. The pigment is distributed in the choroid layer (including the iris, ciliary body, and choroid), and the retinal pigment epithelium (RPE) of the retina [18, 19]. Theoretically, alkaline and lipophilic aromatic compounds could bind to the pigment and accumulate in the tissues where it’s found (such as the eye, colored skin, and colored fat). This affects the drug’s pharmacokinetics and pharmacodynamics processes, in addition to potentially inducing toxicity. For example, hydroxychloroquine, which is a first-line drug used to treating systemic lupus erythematosus, has significant ocular toxicity (including macular degeneration and retinal atrophy). Apparently, this may be attributed to the high affinity of hydroxychloroquine to the pigment, especially considering the high affinity to RPE in the eye [20]. Second, the LE rat’s back skin has pigment, making it an ideal model to study drug accumulation in colored skin [21–24]. Studies have shown that the presence of pigments can greatly affect the distribution of drugs in the hair. In a study of the deposition of codeine in hair, the drug concentration in the pigmented hair of LE rats was six times higher than that in the white hair of SD rats [25].

Analyses and speculations based on pharmacokinetics and tissue distribution studies suggest that there are no significant differences in the metabolic data between the LE and SD rats in non-pigmented tissues. There was no significant difference (P>0.05) in the concentrations of drug-related substances in the plasma and brain homogenates of SD and LE rats (Fig 2). According to the AUC_0-48h_ results calculated using WinNonlin, the pharmacokinetic findings in both plasma and brain homogenates were less than 3.0. Based on these homogenates, there was no significant difference between SD and LE rats (Table 2). Concentrations of drug-related substances in the liver and kidney homogenates of SD and LE rats were significantly different (P<0.05) at 2 and 8 h after drug administration. The ratio of total drug-related substances in the liver homogenates of LE rats was 7.20-fold higher than that of SD rats. Furthermore, the AUC_0-∞_ in the kidney homogenates of LE rats was 7.44-fold higher than that of SD rats. The difference between LE and SD rats in non-pigmented tissues is likely to be caused by the metabolite M8 when generating the metabolite M475-2. A total of 11 metabolites were identified and analyzed through hepatic and renal metabolite characterization in SD rats. M8 was the most predominant metabolite. In the liver, CVL218 undergoes oxidative deamination to form the carboxylic acid metabolite, M8. It’s important to note that the metabolite of M8 conjugated with glucuronic acid is M475-2. However, none of these metabolites was found in the feces, and this suggests that the glucuronic acid metabolite generated by M8 is enriched inside the kidney. Slow excretion of M8 and its metabolites leads to the accumulation of radio-volume, which may also be due to the continuous reabsorption and enterohepatic circulation. We hypothesize that the difference between LE and SD rats in nonpigmented tissues is more likely to be caused by the metabolite, M8, when it generates the metabolite M475-2.

The liver is a blood-rich tissue. The collection of whole blood from the abdominal aorta may affect the M8 in the blood, possibly impacting the results of liver tissue studies. In the case of SD rats, CVL218 undergoes oxidative deamination to form the carboxylic acid metabolite M8 in the liver. The results from this study indicate that CVL218 is rapidly *oxidized* and deaminated in the plasma by MAO-B, following its absorption into the bloodstream [8]. This process produces M8 or *oxidized* and deaminated compounds (M283), which then undergo further oxidation by the liver to yield M8. The presence of a unique inclusion body, the microcylinder, in the *intrarectal* space of the mitochondria was previously reported in various types of cells from rats of the Long-Evans (LE), but was not found in cells of albino rats [26]. Microcylinders are LE rat strain-specific mitochondrial inclusions and consist of protein components, particularly the mitochondrial enzymes CYO and MAO. It can be reasonably deduced that LE rats, which contain a greater quantity of monoamine oxidase, will produce a greater amount of M8 in their liver and kidneys. However, further studies are still required to verify our hypothesis.

It is also suggested that metabolic data between SD and LE rats could be extrapolated from each other [11]. In terms of drug metabolism kinetics, there were no significant differences in the concentrations of [^14^C]CVL218-related substances in the plasma and brain tissue homogenates of LE and SD rats at the same time points. It has been shown that physiologically based pharmacokinetic (PBPK) models based on blood flow data from SD rats can be used to simulate the blood flow data from LE rats [12]. After single oral and intravenous administrations of tolterodine, the pharmacokinetic data in the plasma of LE rats were similar to those in SD rats [13]. When it comes to tissue distribution in non-pigmented tissues, the metabolite spectrum and identification in the brain tissue homogenates of LE and SD rats showed the same spectrum of metabolites with only the original compound. It has been revealed that the total radioactivity of [^14^C]tolterodine-related metabolites in tissue distribution in LE and SD rats is similar [13]. The distribution of endogenous acetylcholinesterase neurons in the dorsal thalamus of LE and SD rats is similar. These two types of rats also have similar immunoreactivity in the intima and adventitia of the cerebral artery cross-section in the brain. There were no differences in [^3^H]arginine vasopressin levels and related renal parameters in the physiological regulation and the distribution of renal antidiuretic hormone receptors in LE and SD rats [14].

The results of tissue distribution in rats are often used to evaluate the amount of radiation exposure in the human body after oral administration of radiolabeled drugs. The detailed calculation is in S1 Appendix.

The radiation energy absorbed by each tissue/organ is related to the energy released by the radiation source and the radiation dose accumulated in each tissue/organ:

D=Ã×Δ×ϕ/m (1)

Where D is the mean absorbed dose (rad); Ã is the accumulated radiation dose (μCi × h); Δ is the average energy released per decay, for [^14^C], Δ = 0.105 rad × g / (μCi × h); ϕ is the fraction of energy emitted in source which is absorbed in each tissue/organ, for [^14^C], ϕ = 1; m is the weight of each tissue/organ (g).

The residence time of radiation dose in each tissue/organ (τ) indicates the ratio of cumulative radiation dose (Ã) to administration radiation dose (A_0_):

$$\tau =Ã/A_{0}$$ (2)

According to Formulas (1) and (2), the absorbed dose of each tissue/organ after oral dose 1 μCi of ^14^C radiolabeled drugs (D/A_0_, unit: rad/ μCi) can be obtained:

$$D/A_{0}=\tau \times 0.105/m$$ (3)

The residence time of radiolabeled drug in each tissue/organ of the human body can be estimated using the tissue distribution results of rats according to the allometric scaling and relative organ mass scaling.


$$\tau ={\mathrm{AUC}}_{0{‐}_{\infty }}\left(\mathrm{rat}‐\mathrm{allometrically}\;\mathrm{scaled}\right)/A_{0}$$ (4)

Based on the tissue distribution results in LE rats that received [^14^C]CLV218, the estimated effective whole-body radiation dose to a male subject weighing 70 kg after a single oral dose of 100 μCi [^14^C]CLV218 was 5.03 mrem.

If the tissue distribution data for the liver and kidney of SD rats (seven times less than LE rats) were replaced, the estimated effective whole-body radiation dose was 4.36 mrem (a reduction of 13.32%). This raises the hypothesis that using the tissue distribution data from SD rats rather than that from LE rats in assessing the human dose in Phase I clinical studies might result in an underestimation of the whole-body effective radiation dose. However, this still needs to be further confirmed.

## 5. Limitations

Comparisons between LE and SD rats using QWBA method were not done in this study. The QWBA method does not allow for the separation and quantification of metabolites. Future studies could introduce additional methods, such as mass spectrometry imaging, to analyze the differences in radio-metabolism-related substances that affect the liver and kidneys of LE and SD rats [27–29].

## 6. Conclusion

Both the OC and QWBA methods revealed that [^14^C]CVL218 could be widely distributed in the tissues of rats. There were no significant differences for most tissues when the two methods were used. There were no differences in the pharmacokinetic data for plasma and brain homogenates between the LE and SD rats. The pharmacokinetic data for metabolism and elimination tissues (liver and kidneys) were seven times higher in LE rats when compared with SD rats. The safety of Using the data from LE rats to estimate the whole-body effective radiation dose of [^14^C]CVL218 for Phase I clinical trials was associated with relatively higher levels of safety.

## Supporting information

S1 Appendix Dose estimation process. (DOCX)

S2 Appendix (XLSX)
