# Supplementary material for: Utility of quantitative whole-body autoradiography (QWBA) and oxidative combustion (OC) analysis in the assessment of tissue distribution of [14C]Mefuparib (CVL218) in SD and LE rats
Source: PLoS One. 2024 Dec 5;19(12):e0315223. doi: 10.1371/journal.pone.0315223 (PMC11620678; doi:10.1371/journal.pone.0315223)
Supplement: S1 Appendix — (DOCX) [file pone.0315223.s001.docx]

The results of tissue distribution in rats are often used to evaluate the amount of radiation exposure in the human body after oral administration of radiolabeled drugs. The calculation process is described as follows:

The radiation energy absorbed by each tissue/organ is related to the energy released by the radiation source and the radiation dose accumulated in each tissue/organ^[1, 2]^:

D = Ã × Δ × ϕ / m (1)

Where D is the mean absorbed dose (rad); Ã is the accumulated radiation dose (μCi × h); Δ is the average energy released per decay, for [^14^C], Δ = 0.105 rad × g / (μCi × h); ϕ is the fraction of energy emitted in source which is absorbed in each tissue/organ, for [^14^C], ϕ = 1; m is the weight of each tissue/organ (g).

The residence time of radiation dose in each tissue/organ (τ) indicates the ratio of cumulative radiation dose (Ã) to administration radiation dose (A_0_):

τ = Ã / A_0_ (2)

According to formulas (1) and (2), the absorbed dose of each tissue/organ after oral dose 1 μCi of ^14^C radiolabeled drugs (D/A_0_, unit: rad/ μCi) can be obtained:

D / A_0_ = τ × 0.105 / m (3)

The residence time of radiolabeled drug in each tissue/organ of the human body can be estimated using the tissue distribution results of rats according to the allometric scaling and relative organ mass scaling.

τ = Ã / A_0_ = AUC_0-∞_(rat - allometrically scaled) / A_0_ (4)

One method of physiological time-scaling is based on relative basal metabolic rates, as modeled by the ratio of the total body masses raised to the 0.25 power^[3, 4]^

t_human_ = t_animal_ × (Body mass_human_ / Body mass_animal_)^0.25^ (5)

Where t_human_ and t_animal_ are the physiological time of human and animal, respectively.

Relative organ mass-scaling assumes that activity uptake and retention are proportional to an organ’s mass relative to its total body mass^[4]^.

%ID Human Organ = %ID Animal Organ × $\frac{{Organ Mass}_{\mathrm{Huma}n}}{{Body Mass}_{\mathrm{Human}}} / \frac{{Organ Mass}_{\mathrm{Animal}}}{{Body Mass}_{\mathrm{Animal}}}$ (6)

Where %ID is the fraction of organ uptake activity to whole body uptake activity.

For example, if the human brain is four times larger as a fraction of its total body mass as the rat brain, then the human brain is assumed to have a percent dose uptake four times greater than the rat brain.

According to (4), (5) and (6), the residence time of radiolabeled drug in each tissue/organ of the human body can be estimated as:

τ = $\frac{\mathrm{AUC}_{0-\infty}(rat o\mathrm{rgan}) \times{(\frac{{Body Mass}_{\mathrm{human}}}{{Body Mass}_{\mathrm{rat}}})}^{0.25} \times\frac{{Organ Mass}_{h\mathrm{uma}n}}{{Body Mass}_{h\mathrm{uman}}} / \frac{{Organ Mass}_{\mathrm{rat}}}{{Body Mass}_{r\mathrm{at}}}}{A_{0}}$ (7)

Where AUC_0-∞_ (rat organ) were calculated using experimental data; Organ Mass_rat_ was collected on a per-gram basis in the study; Organ Mass_human_ can refer to the literature^[2]^; A_0_ was dose for rat, the unit is consistent with AUC_0-∞_ (rat organ).

The estimated τ and the designed dose A_0_ can be used to calculate the D in each tissue/organ of the human body from formula (3) after dosing.

$E=\sum W_{T}D_{T}$ (8)

Where E was the effective whole-body radiation dose; W_T_ was the weight coefficient for each tissue/organ, which can refer to the literature^[5]^. D_T_ was the effective radiation dose of each tissue/organ of the human body.

Based on above process, the effective whole-body radiation dose to a male subject weighing 70 kg after a single oral dose of 100 µCi [^14^C]CLV218 can be evaluated.

References

[1] M.G. Stabin. MIRDOSE: personal computer software for internal dose assessment in nuclear medicine. J Nucl Med. 1996 Mar, 37(3): 538-546. https://pubmed.ncbi.nlm.nih.gov/8772664/

[2] R. Loevinger, T.F. Budinger, E.E. Watson. MIRD Primer for Absorbed Dose Calculations, Revised Edition. Prepared by the Society of Nuclear Medicine, New York, USA, 1991.

[3] J. Gabrielsson, D. Weiner. Pharmacokinetic and Pharmacodynamic Data Analysis: Concepts and Applications, Third Edition. Swedish Pharmaceutical Press, Stockholm, Sweden, 2001: 164.

[4] R.B. Sparks, B. Aydogan. Comparison of the effectiveness of some common animal data scaling techniques in estimating human radiation dose. Sixth International Radiopharmaceutical Dosimetry Symposium, Orise 99-0164, 1999: 705-716. <https://www.osti.gov/biblio/684479>

[5] Annals of the ICRP (2007), Recommendations of the interational commission on radiological protection. ICRP Publication 103. http://www.icrp.org/publication.asp?id= ICRP Publication.
